# Prevalence and Impact on Stroke in Patients Receiving Maintenance Hemodialysis versus Peritoneal Dialysis: A Prospective Observational Study

**DOI:** 10.1371/journal.pone.0140887

**Published:** 2015-10-20

**Authors:** Junzhou Fu, Jun Huang, Ming Lei, Zhengmao Luo, Xianyang Zhong, Yuanhang Huang, Hong Zhang, Riguang Liu, Junrong Tong, Feng He

**Affiliations:** 1 Department of Nephrology, Guangzhou First People's Hospital, Guangzhou Medical University, Guangzhou 510180, China; 2 Graduate School of Southern Medical University, Guangzhou 510515, China; 3 Department of Nephrology, General Hospital of Guangzhou Military Command of PLA, Guangzhou 510010, China; University of Utah School of Medicine, UNITED STATES

## Abstract

**Background:**

Patients undergoing maintenance dialysis are at increased risk of stroke, however, less is known about the prevalence and impact on stroke in the patients.

**Methods:**

In this prospective cohort study, 590 patients undergoing hemodialysis (HD; n = 285) or peritoneal dialysis (PD; n = 305) from January 1, 2008 to December 31, 2012 were recruited. Baseline demographic, clinical, and laboratory data were collected. Timeline incidence data were analyzed using a Poisson model. The Cox proportional hazards regression assessed adjusted differences in stroke risk, a multivariate analysis was also performed.

**Results:**

62 strokes occurred during 1258 total patient-years of follow-up. Stroke occurred at a rate of 49.2/1,000 patient-years with a predominance in HD patients compared with PD patients (74.0 vs. 31.8/1,000 patient-years). The cumulative hazard of developing stroke was significantly higher in HD patients (hazard ratio [HR], 1.75; 95% confidence interval [CI], 1.15–3.62; *p* = 0.046) after adjusting for potential confounders. HD patients had an increased risk of ischemic stroke (HR, 2.62; 95% CI, 1.56–4.58; *p* = 0.002). The risk of hemorrhagic stroke was not significantly different between PD and HD patients. On multivariate Cox analysis, risk factors of stroke in both HD and PD patients were older age, diabetes, and cardiovascular disease. Other independent risk factors of stroke were lower albumin-corrected calcium in HD patients and higher triglycerides in PD patients.

**Conclusions:**

Patients undergoing PD were less likely to develop ischemic stroke than those undergoing HD. Comprehensive control of diabetes, cardiovascular disease, calcium-phosphorus metabolism, and triglyceride levels may be useful preventive strategies for stroke in dialysis patients.

## Introduction

The global incidence of end-stage renal disease (ESRD) has been increasing steadily. Hemodialysis (HD) or peritoneal dialysis (PD) has been widely accepted for treatment in patients with ERSD [1]. Cardiovascular disease (CVD) is the leading cause of death in these patients, accounting for 40%–50% of all-cause mortality [2]. Stroke is the leading cause of cardiovascular mortality in patients with ESRD, and as the second and third leading causes of death in the United States (US) and United Kingdom, respectively [3, 4]. Previous studies in the US and Japan reported 2- to 10-fold increased risks of stroke in dialysis patients compared to the general population [5, 6]. This propensity for stroke has been attributed to their higher prevalence of factors recognized as risk factors for stroke in the general population, such as hypertension and diabetes mellitus, plus the presence of factors specific to patients receiving dialysis for ESRD, such as accelerated calcific arteriosclerosis, effects of uremic toxins, dialysis techniques, vascular access, and use of anticoagulants to maintain flow in the extracorporeal circuit [7, 8]. The mortality rate of stroke in dialysis patients was reported to be nearly three times higher than that of patients not receiving dialysis [9]. Most studies of patients with ESRD have focused on HD patients, and less is known about the incidence, relative risks, and subtypes of stroke in PD patients.

We designed the present prospective observational cohort study to estimate the incidence rates for different subtypes of stroke in patients undergoing HD or PD, and to identify prognostic risk factors of stroke events associated with each dialysis modality.

## Participants and Methods

### Ethics statement

The study protocol was approved by the Clinical Research Ethics Committee of Guangzhou First People's Hospital (Gaungzhou, China) and the Clinical Research Ethics Committee of General Hospital of Guangzhou Military Command of PLA (Gaungzhou, China). All patients provided written informed consent before study entry.

### Participants

Patients were recruited from two dialysis centers (Guangzhou First People's Hospital and General Hospital of Guangzhou Military Command of PLA) between January 1, 2008 and December 30, 2012. The follow-up period extended to December 31, 2013. The inclusion criteria for this study were all patients ≥18 years old who received maintenance HD or PD therapy for more than 3 months, except those who had ever received a kidney transplant prior to dialysis, had malignant disease, or refused to give written consent. Patients in whom the dialysis modality changed during the study period were classified as HD or PD according to their initial treatment modality.

### Study Protocol

This was a prospective observational cohort study. Baseline demographic data included age, gender, 24-h urine output, dry weight, primary cause of ESRD, history of diabetes, hypertension, or CVD, and biochemical parameters, including hemoglobin, serum albumin, albumin-corrected calcium, serum phosphorus, total cholesterol, total triglycerides, serum high sensitive C-reactive protein (hs-CRP), serum uric acid, serum urea nitrogen, serum creatinine, total Kt/V, and residual kidney function (RKF). These data were obtained during the first 1–3 months of dialysis. All parameters were measured in the central laboratory of the two hospitals. Medicine usage data were also collected from the patients’ files.

The major outcome of the study was stroke, which was defined as a focal neurological deficit of cerebrovascular persisting for >24 h that was diagnosed as an ischemic or hemorrhagic stroke by computed tomography (CT) or magnetic resonance imaging (MRI). They included the International Classification of Diseases, Ninth Revision, Clinical Modification (ICD-9-CM) diagnosis codes of 433.xx, 434.xx, and 436.xx for infarction and 430.xx, 431.xx, and 432.xx for hemorrhage. The quality of diagnosis data fulfilled the criteria of the ideal stroke incidence study suggested by Sudlow and Warlow [10]. In addition, the diagnosis of diabetes at the initiation of dialysis was based on diagnostic criteria from the American Diabetes Association [11]. Hypertension was recorded if the patient was taking any antihypertensive drug or had two separate blood pressure measurements ≥140/90 mmHg. CVD was defined that includes myocardial infarction, atherosclerotic heart disease, cardiomyopathy, cardiac arrhythmia, cardiac arrest, congestive heart failure, cerebrovascular accident, ischemic brain damage, anoxic encephalopathy, and peripheral vascular disease [12]. Body mass index (BMI) was calculated as weight (kg) divided by height (m) squared. Residual kidney function (RKF) and total Kt/V were calculated using PD adequest software 2.0 (Baxter Healthcare Ltd). The RKF, in milliliters per minute per 1.73 m^2^, was estimated from mean values of creatinine clearance and urea clearance and adjusted for body surface area [12].

### Statistical Analysis

Summary statistics were presented as percentages for categorical data, mean ± standard deviation for approximately normally distributed continuous variables, and median (interquartile range) for skewed continuous variables. According to the primary dialysis modality, patients were divided into two groups: HD and PD. According to stroke events, patients were also divided into two groups: stroke and non-stroke. Characteristic differences between two groups were tested by using the chi-square test for categorical variables, Student’s t-test for approximately normally distributed continuous variables, and the non-parametric Mann-Whitney test for skewed continuous variables. Timeline incidence data were analyzed using a Poisson model. Time-to-event analysis of stroke events was performed using Kaplan-Meier survival curves, the Log-Rank test and the Cox proportional hazards model for the HD group compared to the PD group. Multivariate models were constructed sequentially by initially using only the group, then adding demographic characteristics (age at enrollment and gender), followed by adding comorbidities (diabetes, hypertension and cardiovascular disease), and finally by adding laboratory values (albumin-corrected calcium, total triglycerides, hs-CRP, 24-h urine output, and RKF). Cox regression analysis was used to evaluate the risk factors for the presence of stroke in the total dialysis population, HD patients, or PD patients at baseline. Two different approaches were applied: (1) univariate Cox regression analysis of the occurrence of stroke using the following variables: age, diabetes, hypertension, cardiovascular disease, albumin-corrected calcium, total triglycerides, 24-h urine output, RKF, and aspirin and clopidogrel use, and (2) multivariate Cox regression analysis modeling of covariates using a backward stepwise selection procedure (entry: *p* ≤0.05; removal: *p* >0.1; the selection criterion was derived from acquiesce in SPSS software system, as well as the estimated clinical importance). Furthermore, Cox regression models were used to evaluate the relationship between total triglycerides tertiles with stroke, initially without adjustment, and subsequently adjusting for several groups of covariates. The multivariate Cox regression model were constructed using eligible covariates that demonstrated significant or near significant association with stroke (*p* < 0.2) on bivariable analysis or for importance of clinical concern. Statistical significance was defined as *p* <0.05 using two-tailed tests. Statistical analyses were performed using SPSS 17.0 for Windows (SPSS, Chicago, IL, USA).

## Results

### Baseline Cohort Characteristics

The demographic and clinical characteristics of the study cohort categorized by dialysis modality are shown in Table 1. A total of 590 eligible patients (mean age 54.46±15.65 years, 61.8% male) undergoing HD (n = 285) or PD (n = 305) were enrolled in this study. The median follow-up duration was 32.5 months (range: 3–71.8). The primary cause of ESRD was glomerular disease (261 patients; 44.2%), followed by diabetic nephropathy (146 patients; 24.7%) and hypertension (96 patients; 16.2%). Compared to PD patients, HD patients more frequently had diabetes and CVD and presented with an older age and a higher hs-CRP, but lower total triglycerides, Kt/V, 24-h urine output, and RKF. During the study period, 91 (15.4%) patients underwent renal transplantation, 30 (5.1%) patients were transferred to other centers, 17 (3.0%) patients were lost to follow-up, 4 (0.6%) patients declined further treatment, and the remaining 449 (75.9%) patients continued to be followed up.

**Table 1:** Baseline characteristics of dialysis patients.

Variables	Total(n = 590)	HD group(n = 285)	PD group(n = 305)	*p* value
Demographics				
Gender (male; n, %)	364 (61.7%)	185 (64.9%)	179 (58.7%)	0.075
Age (y)	54.46±15.65	57.84±16.15	52.67±14.85	< 0.001
Body mass index (kg/m^2^)	21.95±3.46	22.87±3.79	21.56±2.98	0.160
Dry weight (kg)	57.72±10.77	58.68±12.20	57.35±9.28	0.920
Comorbidities				
Diabetes (n, %)	156 (26.4%)	94 (33.0%)	62 (20.3%)	< 0.001
Hypertension (n, %)	376 (63.7%)	183 (64.2%)	193 (63.3%)	0.597
Systolic BP (mmHg)	136.6±21.8	137.0±22.3	136.3±19.7	0.519
Diastolic BP (mmHg)	85.0±14.1	85.5±13.8	84.9±14.4	0.335
Cardiovascular disease (n, %)	186 (31.5%)	102 (35.7%)	84 (27.5%)	0.013
Etiology of renal disease				
Chronic glomerulonephritis (n, %)	261 (44.2%)	115 (40.1%)	146 (47.8%)	0.066
Diabetic nephropathy (n, %)	146 (24.7%)	88 (30.8%)	58 (19.0%)	0.001
Hypertensive (n, %)	96 (16.2%)	36 (12.6%)	60 (19.7%)	0.013
Other/unknown (n, %)	89 (15.1%)	46 (16.2%)	43 (14.1%)	0.062
Laboratory variables				
Hemoglobin (g/L)	97.57±18.04	96.76±20.28	97.77±16.05	0.150
Serum albumin (g/L)	36.85±4.61	36.37±5.10	37.30±4.07	0.065
Albumin-corrected calcium (mmol/L)	2.45±0.37	2.43±0.39	2.46±0.36	0.830
Serum phosphorus (mmol/L)	1.43±0.47	1.40±0.39	1.47±0.54	0.520
Total triglycerides (mmol/L)	1.74±0.94	1.69±0.93	1.79±0.98	0.045
Total cholesterol (mmol/L)	4.98±1.12	4.96±1.03	5.02±1.10	0. 490
HDL-C (mmol/L)	1.25±0.59	1.26±0.57	1.22±0.58	0.337
LDL-C (mmol/L)	2.96±1.32	2.93±1.36	2.99±1.07	0.216
hs-CRP (mg/L)	1.89 (0.65–7.11)	2.02 (0.72–7.35)	1.70 (0.63–6.42)	0.001
Serum uric acid (μmol/L)	439.00±127.00	444.00±90.00	435.00±115.00	0.460
Serum urea nitrogen (mmol/L)	15.9 (7.7–22.7)	15.8 (7.9–23.5)	16.0 (8.0–22.1)	0.577
Serum creatinine (μmol/L)	745±321	725±305	761±312	0.070
Total Kt/V	2.29±0.57	2.16±0.54	2.40±0.69	0.010
24-h urine output (mL)	570 (0–1250)	380 (0–800)	810 (400–1300)	< 0.001
RKF (mL/min/1.73 m^2^)	2.87±2.35	1.96±1.65	3.59±2.86	0.001
Treatment				
Aspirin	260 (44.1%)	125 (43.9%)	135 (44.3%)	0.16
Clopidogrel	97 (16.4%)	48 (16.8%)	49 (16.1%)	0.22

Values for continuous variables are mean±standard deviation or median (interquartile range). HD, hemodialysis; PD, peritoneal dialysis; SBP, systolic blood pressure; DBP, diastolic blood pressure; HDL-C, high-density lipoprotein cholesterol; LDL-C, low-density lipoprotein cholesterol; hs-CRP, high sensitivity C-reactive protein; RKF, residual kidney function.

### Characteristics of Stroke Patients

A total of 62 stroke events (10.5%) occurred in all patients, including 39 (62.9%) ischemic strokes and 23 (37.1%) hemorrhagic strokes (Table 2). There were 38 (61.3%) events in the HD group and 24 (38.7%) events in the PD group. The ischemic stroke incidence rate was higher in the HD group than in the PD group (8.7% vs. 4.6%; *p* = 0.039). The overall incidence rate of stroke was 49.2/1,000 patient-years for all patients, 74.0/1,000 patient-years in the HD group, and 31.8/1,000 patient-years in the PD group (*p*<0.001).

**Table 2:** Case rates by type of stroke in the study population.

Type of stroke	Total(n = 62)	HD group(n = 38)	PD group(n = 24)	*p* value
Ischemic	39	25 (25/38)	14 (14/24)	0.039
Hemorrhagic	23	13 (13/38)	10 (10/24)	0.054

HD, hemodialysis; PD, peritoneal dialysis.

**Fig 1:**
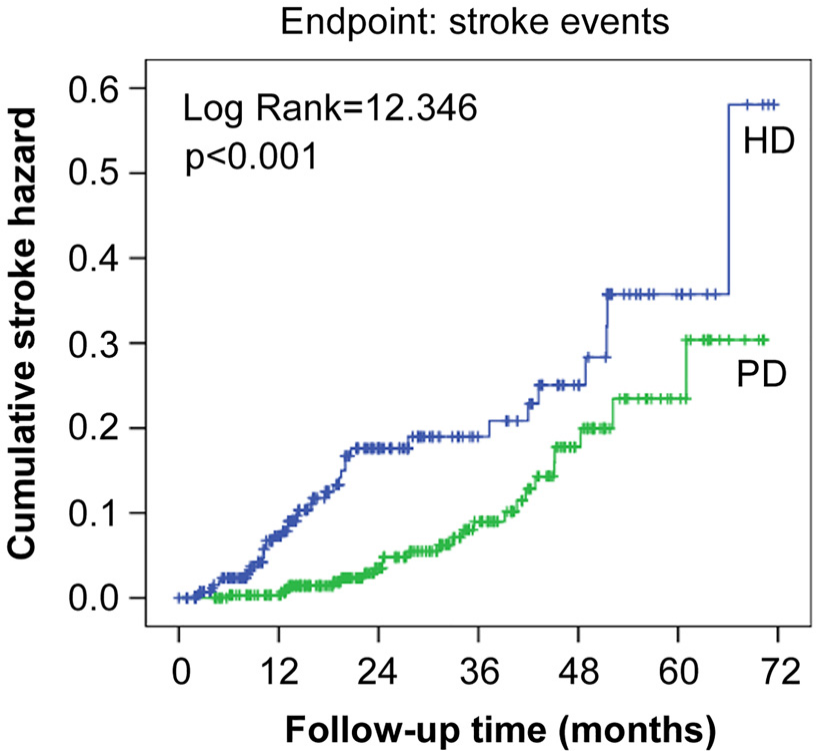
Cumulative hazard of stroke events in hemodialysis and peritoneal dialysis groups. The hazard ratio in the hemodialysis group compared to the peritoneal dialysis group was 2.43 (95% confidence interval, 1.45–4.06). HD, hemodialysis; PD, peritoneal dialysis.

**Fig 2:**
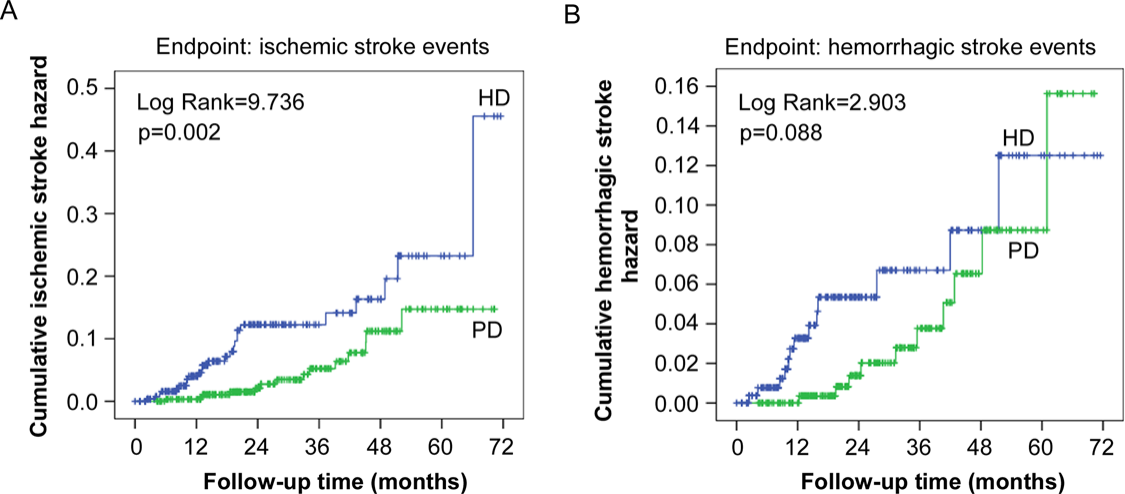
Cumulative hazard of ischemic and hemorrhagic stroke events in hemodialysis and peritoneal dialysis groups. (A) Cumulative hazard of ischemic stroke. The hazard ratio in the hemodialysis group compared to the peritoneal dialysis group was 2.62 (95% confidence interval, 1.56–4.58). (B) Cumulative hazard of hemorrhagic stroke. HD, hemodialysis; PD, peritoneal dialysis.

The characteristics of stroke patients are shown in Table 3. Not surprisingly, compared to non-stroke patients, stroke patients had an older age (65.09±11.27 vs. 50.53±16.30; *p*<0.001), total triglycerides (2.12±1.09 vs. 1.62±0.87; *p* = 0.004), and hs-CRP (2.11 [0.68–7.37] vs. 1.68 [0.63–6.40]; *p*<0.001). They also had a lower albumin-corrected calcium (2.29±0.39 vs. 2.41±0.35; *p* = 0.030), 24-h urine output (536±295 vs. 680±342; *p* = 0.043), and RKF (2.05±1.26 vs. 3.57±2.52; *p* = 0.002). In addition, stroke patients more frequently had the comorbidities of diabetes, hypertension, and cardiovascular disease, and they were more commonly being treated with aspirin and clopidogrel. Furthermore, the cumulative hazard of developing stroke was significantly higher in the HD group than in the PD group (hazard ratio [HR], 2.43; 95% confidence interval [CI], 1.45–4.06; *p*<0.001) (Fig 1). After adjusting for various potential confounders, there was still a significant association between HD and the risk of stroke (HR, 1.75; 95% CI, 1.15–3.62; *p* = 0.046) (Table 4). Moreover, compared to PD patients, HD patients had a higher risk of ischemic stroke based on Kaplan-Meier curves and Cox regression analysis (HR, 2.62; 95% CI, 1.56–4.58; *p* = 0.002) (Fig 2). However, the risk of hemorrhagic stroke did not differ between the two groups.

**Table 3:** Comparative baseline characteristics of patients with and without stroke.

Variables	Stroke(n = 62)	Non-stroke(n = 528)	*p* value
Demographics			
Gender (male, n, %)	37 (59.7%)	327 (61.9%)	0.722
Age (y)	65.09±11.27	50.53±16.30	< 0.001
Body mass index (kg/m^2^)	22.33±3.17	21.91±3.50	0.388
Dry weight (kg)	58.77±12.35	57.32±10.36	0.961
Comorbidities			
Diabetes (n, %)	36 (58.1%)	120 (22.7%)	< 0.001
Hypertension (n, %)	45 (72.5%)	331 (62.7%)	0.001
Systolic BP (mmHg)	138.0±20.3	136.5±19.5	0.092
Diastolic BP (mmHg)	87.9±15.4	84.6±14.2	0.005
Cardiovascular disease (n, %)	38 (61.2%)	148 (28.0%)	< 0.001
Etiology of renal disease			
Chronic glomerulonephritis (n, %)	17 (27.5%)	244 (46.2%)	0.013
Diabetic nephropathy (n, %)	28 (45.7%)	118 (22.3%)	< 0.001
Hypertensive (n, %)	9 (14.5%)	87 (16.5%)	0.155
Other/unknown (n, %)	8 (12.3%)	81 (15.3%)	0.054
Laboratory variables			
Hemoglobin (g/L)	97.64±19.29	97.61±18.36	0.915
Serum albumin (g/L)	36.26±5.16	37.31±4.54	0.124
Albumin-corrected calcium (mmol/L)	2.29±0.39	2.48±0.35	0.038
Serum phosphorus (mmol/L)	1.49±0.57	1.45±0.53	0.135
Total triglycerides (mmol/L)	2.12±1.09	1.70±0.90	0.010
Total cholesterol (mmol/L)	5.11±1.17	4.96±1.09	0.862
HDL-C (mmol/L)	1.21±0.53	1.27±0.61	0.196
LDL-C (mmol/L)	3.03±1.39	2.95±1.20	0.092
hs-CRP (mg/L)	2.11 (0.68–7.37)	1.68 (0.63–6.40)	< 0.001
Serum uric acid (μmol/L)	417.35±135.38	430.98±95.00	0.310
Serum urea nitrogen (mmol/L)	16.2 (7.7–23.9)	15.8 (7.6–22.8)	0.648
Serum creatinine (μmol/L)	753±321	746±308	0.136
Total Kt/V	2.25±0.57	2.38±0.64	0.224
24-h urine output (mL)	536±295	680±342	0.043
RKF (mL/min/1.73 m^2^)	2.05±1.26	3.57±2.52	0.002
Treatment			
Aspirin	41(66.5%)	219(41.5%)	0.001
Clopidogrel	20(32.3%)	77(14.6%)	0.026

Values for continuous variables are mean±standard deviation or median (interquartile range). HD, hemodialysis; PD, peritoneal dialysis; SBP, systolic blood pressure; DBP, diastolic blood pressure; HDL-C, high-density lipoprotein cholesterol; LDL-C, low-density lipoprotein cholesterol; hs-CRP, high sensitivity C-reactive protein; RKF, residual kidney function.

**Table 4:** Hazard ratio of stroke in hemodialysis versus peritoneal dialysis groups.

	HR	95% CI	*p* Value
Unadjusted	2.43	1.45–4.06	0.001
Model 1 ^a^	2.14	1.43–3.91	0.013
Model 2 ^b^	1.96	1.34–3.76	0.030
Model 3 ^c^	1.75	1.15–3.62	0.046

^a^ adjusted for age and sex.

^b^ adjusted for model 1 covariates and diabetes, hypertension and cardiovascular disease.

^c^ adjusted for model 2 covariates and albumin-corrected calcium, total triglycerides, hs-CRP, 24-h urine output and residual kidney function.

HR, hazard ratio; CI, confidence interval.

### Risk Factors for Stroke

Clinical and laboratory variables that were statistically different in Table 3 were included in the Cox regression analysis. Table 5 shows the risk factors for stroke for all patients. The multivariate analysis model identified these factors as being independently associated with an increased risk of stroke: older age (HR, 1.05; 95% CI, 1.02–1.09; *p* = 0.003), diabetes (HR, 1.98; 95% CI, 1.31–3.46; *p* = 0.001), CVD (HR, 2.06; 95% CI, 1.62–3.05; *p*<0.001), higher triglycerides (HR, 1.20; 95% CI, 1.08–1.58; *p* = 0.034), and HD (with PD as the reference; HR, 2.03; 95% CI, 1.46–3.89; *p* = 0.005). The predictors of stroke for HD and PD patients are shown in Table 6 and Table 7, respectively. In addition to the common stroke risk factors of older age, diabetes, and CVD that were predictors for both groups, lower albumin-corrected calcium (HR, 0.92; 95% CI, 0.74–0.96; *p* = 0.035) was an independent prognostic predictor for stroke in HD patients, whereas higher triglycerides (HR, 1.28; 95% CI, 1.07–1.62; *p* = 0.016) was an independent risk factor for stroke in PD patients. Furthermore, we found a J-shaped effect of total triglycerides with stroke (S1 Table, S1 Fig).

**Table 5:** Risk factors for stroke in all dialysis patients.

Risk factors ^a^	Univariate Analysis		Multivariate Analysis	
	HR (95% CI)	*p* value	HR (95% CI)	*p* value
Age (per 1 year increase)	1.07 (1.05–1.09)	<0.001	1.05 (1.02–1.09)	0.003
Diabetes (yes/no)	3.11 (1.48–4.80)	<0.001	1.98 (1.31–3.46)	0.001
Hypertension (yes/no)	1.88 (1.03–3.42)	0.038	—	—
Cardiovascular disease (yes/no)	3.62 (2.19–5.96)	<0.001	2.06 (1.62–3.05)	<0.001
Albumin-corrected calcium (per 1 mmol/L increase)	0.82 (0.61–1.04)	0.060	—	—
Total triglycerides (per 1 mmol/L increase)	1.36 (1.11–1.66)	0.003	1.20 (1.08–1.58)	0.034
24-h urine output (mL)	0.98 (0.96–1.03)	0.172	—	—
RKF (mL/min/1.73 m^2^)	0.88 (0.69–1.24)	0.065	—	—
Aspirin (yes/no)	0.92 (0.88–1.16)	0.082	—	—
Clopidogrel (yes/no)	0.90 (0.76–1.09)	0.092	—	—
Hemodialysis ^b^	2.43 (1.45–4.06)	0.001	2.03 (1.46–3.89)	0.005

^a^ Factors that exhibited a difference in Table 2 were included in the Cox proportional risk regression analysis.

^b^ Reference is the peritoneal dialysis group. HR, hazard ratio; CI, confidence interval; RKF, residual kidney function.

**Table 6:** Risk factors for stroke in hemodialysis patients.

Risk factors ^a^	Univariate Analysis		Multivariate Analysis	
	HR (95%CI)	*p* value	HR (95%CI)	*p* value
Age (per 1 year increase)	1.07 (1.02–1.09)	0.001	1.06 (1.03–1.08)	0.004
Diabetes (yes/no)	3.89 (1.99–7.64)	<0.001	2.19 (1.68–5.02)	<0.001
Hypertension (yes/no)	2.16 (0.90–5.18)	0.084	—	—
Cardiovascular disease (yes/no)	4.07 (2.55–7.28)	<0.001	2.46 (1.92–4.96)	<0.001
Albumin-corrected calcium (per 1 mmol/L increase)	0.89 (0.62–0.96)	0.006	0.92 (0.74–0.96)	0.035
Total triglycerides (per 1 mmol/L increase)	1.29 (0.94–1.77)	0.115	—	—
24-h urine output (mL)	0.99 (0.98–1.01)	0.298	—	—
RKF (mL/min/1.73 m^2^)	0.91 (0.72–1.29)	0.106	—	—
Aspirin (yes/no)	0.90 (0.78–1.15)	0.099	—	—
Clopidogrel (yes/no)	0.88 (0.64–1.12)	0.113	—	—

^a^ Factors that exhibited a difference in Table 2 were included in the Cox proportional risk regression analysis. HR, hazard ratio; CI, confidence interval; RKF, residual kidney function.

**Table 7:** Risk factors for stroke in peritoneal dialysis patients.

Risk factors ^a^	Univariate Analysis		Multivariate Analysis	
	HR (95% CI)	*p* value	HR (95% CI)	*p* value
Age (per 1 year increase)	1.06 (1.03–1.09)	<0.001	1.04 (1.02–1.08)	0.003
Diabetes (yes/no)	3.01 (1.51–5.61)	0.003	1.86 (1.35–4.22)	0.004
Hypertension (yes/no)	1.30 (0.55–3.07)	0.544	—	—
Cardiovascular disease (yes/no)	3.49 (2.27–6.05)	<0.001	2.02 (1.58–3.46)	0.002
Albumin-corrected calcium (per 1 mmol/L increase)	0.77 (0.50–1.43)	0.157	—	—
Total triglycerides (per 1 mmol/L increase)	1.42 (1.09–1.86)	0.001	1.28 (1.07–1.62)	0.016
24-h urine output (mL)	0.98 (0.97–1.04)	0.326	—	—
RKF (mL/min/1.73 m^2^)	0.92 (0.71–1.35)	0.158	—	—
Aspirin (yes/no)	0.88 (0.76–1.18)	0.010	—	—
Clopidogrel (yes/no)	0.92 (0.81–1.10)	0.106	—	—

^a^ Factors that exhibited a difference in Table 2 were included in the Cox proportional risk regression analysis. HR, hazard ratio; CI, confidence interval; RKF, residual kidney function.

## Discussion

This prospective observational cohort study offers a detailed evaluation of the epidemiology, clinical features, and risk factors of stroke in dialysis patients. The main findings were that (i) HD patients had a higher incidence and cumulative hazard of developing stroke, especially the ischemic subtype, compared to PD patients; and (ii) the risk factors for stroke were older age, diabetes, and CVD in both HD and PD patients, lower albumin-corrected calcium in HD patients, and higher triglycerides in PD patients.

Despite the known high prevalence of CVD in the dialysis population, cerebrovascular disease and its stroke subtypes have been poorly studied. The National Health Insurance Research Database (NHIRD) study [13] showed that HD and PD patients had higher incidences of hospitalized ischemic stroke (102.6 and 100.1/10,000 person-years) and hemorrhagic stroke (74.7 and 59.4/10,000 person-years) compared to an age-and sex-matched reference general population cohort (42.4 and 13.0/10,000 person-years, respectively). Power et al showed that stroke incidents occurred at a rate of 14.9/1,000 patient years (95% CI, 12.2–17.9) in HD patients [14]. Perales et al found that the prevalence of ischemic stroke was high at the commencement of dialysis (HD or PD), and its incidence increased during follow-up to an incidence of 24.1/1,000 patient-years [15]. For comparison to our population, we collected data regarding the incidence of stroke as diagnosed by computed tomography or magnetic resonance imaging during follow-up. We found that the overall stroke rate was 49.2/1,000 patient-years and that the stroke rate of 74.0/1,000 patient-years in HD patients was higher than of the stroke rate of 31.8/1,000 patient-years in PD patients.

We report an overall stroke incidence markedly higher than the upper literatures [13–15], but in keeping with the US rates [16, 17] that a high incidence of stroke at 49/1,000 patient years in their HD and peritoneal dialysis population, which probably we applied a standard to diagnosed stroke by that having focal neurological deficit persisting for >24 h using computed tomography or magnetic resonance imaging, according to the opinion of the attending neurologist, whereas not the relative strict standard of the first hospitalization because of stroke, which might lead to miss some cases.

An important finding in the present study was our confirmation that HD patients had a significantly higher overall risk of stroke compared to PD patients (HR, 2.43; 95% CI, 1.45–4.06). This was applicable for ischemic stroke (HR, 2.62; 95% CI, 1.56–4.58), but there was no difference in the risk of hemorrhagic stroke between the two groups. These results agree with those of Power et al’s [10] large-scale randomized trial, which showed that HD patients had an extraordinarily high risk of stroke, with a predominance of ischemic compared to hemorrhagic subtypes (11.2 vs. 3.7/1,000 patient-years). Although the NHIRD study found that PD patients had a lower risk of hemorrhagic stroke than HD patients (HR, 0.75; 95% CI, 0.58–0.96), and there was no significant difference in risks of ischemic stroke between PD and HD patients after adjusting for all potential confounders [13], HD patients presented with a higher risk of stroke. Of note, that there may be possible influence factors for the higher risk of stroke in HD patients than PD patients cannot be ignored. In the present study, PD patients had better baseline parameters than HD patients, who were more likely to exhibit characteristics that could increase the risk of stroke, such as an older age; the presence of comorbidities such as diabetes, hypertension, and CVD; and worse nutritional status and residual renal function.

Furthermore, we found that the independent risk factors for stroke in both HD and PD patients were older age, diabetes and CVD, which were nonmodifiable risk factors for stroke reported in a number of previous studies [15, 16, 18–20]. Thus, although different dialysis modalities may have varied clinical effects in terms of the development of stroke, comprehensive control of diabetes and CVD remains the basic strategy for management of the aforementioned risk factors. Consistently, the aging process is an important risk factor for stroke in the general population, especially in dialysis patients [11]. Iseki and colleagues [21] and Power et al [14] reported that the mean age at hemorrhagic stroke onset was lower than the age at ischemic stroke onset in dialysis patients. Importantly, in evaluating laboratory variables in the present study, we found that stroke patients had lower levels of albumin-corrected calcium and it as a protective factor for stroke incidence in HD patients, which in keeping with the report of Perales et al [15], but the result of Slinin et al [22] that higher calcium concentration was predictive of de novo stroke. There is a paucity of data regarding the associations between disorders of calcium-phosphorus metabolism and the presentation of adverse stroke events in dialysis patients.

Moreover, we found that the hazard ratio for stroke per 1-mmol/L increase in total triglycerides was 1.28 (95% CI, 1.07–1.62) for PD patients. Triglycerides are especially concerning in dialysis patients, who are more likely than the general population to develop CVD [23]. Despite the modest increase in serum triglyceride levels in dialysis patients, which are usually in the range of 200 to 300 mg/dL, their contribution to atherosclerosis cannot be underestimated [24]. Several possible explanations have been proposed for the increased risk of stroke in PD patients with higher triglyceride levels. First, PD patients require a diet higher in protein than those on a hemodialysis diet because protein is removed through the peritoneal membrane with every dialysis exchange [25]. As a result of the dietary increase in protein, PD patients also consume more fat than hemodialysis patients. Second, triglycerides may also increase more in PD patients than hemodialysis patients because PD patients receive glucose in their PD dialysate which can be stored as fat [26]. PD patients need to monitor their fat intake a little more closely. Some proteins are also lost during hemodialysis, but not as much with PD. Thus, we tentatively conclude that PD patients with higher triglycerides levels appear to be at higher risk of stroke than those in HD patients, and aim to decreasing serum triglyceride levels in PD patients will also show beneficial clinical effects of reducing stroke and CVD events.

The limitations of this study should be considered. First, we failed to randomly allocate ESRD patients to HD or PD therapy, which may have contributed to indication bias. Second, some important variables and information that might affect the risk of stroke, such as smoking, psychosocial stress, and alcohol consumption, were not available for us to incorporate into the study. Third, the risk was calculated on the basis of comorbidities, biological factors, and treatment only at baseline. The variability in these characteristics over time was not determined or accounted for, although this is a limitation that was present in all other studies published to date.

In conclusion, we found that patients undergoing HD had a significantly higher risk of stroke, with an adjusted hazard ratio of 1.75 (1.15–3.62) compared to patients undergoing PD. This increased risk was especially apparent for the subtype of ischemic stroke. The risk factors for stroke for all dialysis patients were older age, diabetes, and CVD, whereas lower albumin-corrected calcium was a predictor in HD patients and higher triglyceride levels were associated with an increased risk of stroke in PD patients. Although different dialysis modalities involve different mechanisms of pathophysiology, comprehensive control of diabetes, CVD, calcium-phosphorus metabolism, and triglyceride levels may be an effective preventive strategy for stroke in dialysis patients.

## Supporting Information

S1 Fig The relationship for total triglycerides with stroke. Compared with the Tertile II (≥1.09-≤1.48 mmol/L) as a reference, hazard ratios of stroke were 1.12 (95% confidence interval [CI], 0.88–1.41) for Tertile I (<1.09 mmol/L), 1.04 (95% CI, 0.90–1.28) for Tertile III (>1.48-≤1.90 mmol/L), and 1.53 (95% CI, 1.15–2.18) for Tertile IV (>1.90 mmol/L). Adjusted for age, sex, BMI, comorbidities, hemoglobin, serum albumin, albumin-corrected calcium, total cholesterol, hs-CRP, 24-h urine output, residual kidney function.
(TIF)

S1 Table The Cox regression analysis of triglycerides for stroke in all dialysis patients. (DOCX)
