# Supplementary figures and images for: Prevalence and Impact on Stroke in Patients Receiving Maintenance Hemodialysis versus Peritoneal Dialysis: A Prospective Observational Study
Source: PLoS One. 2015 Oct 20;10(10):e0140887. doi: 10.1371/journal.pone.0140887 (PMC4617449; doi:10.1371/journal.pone.0140887)

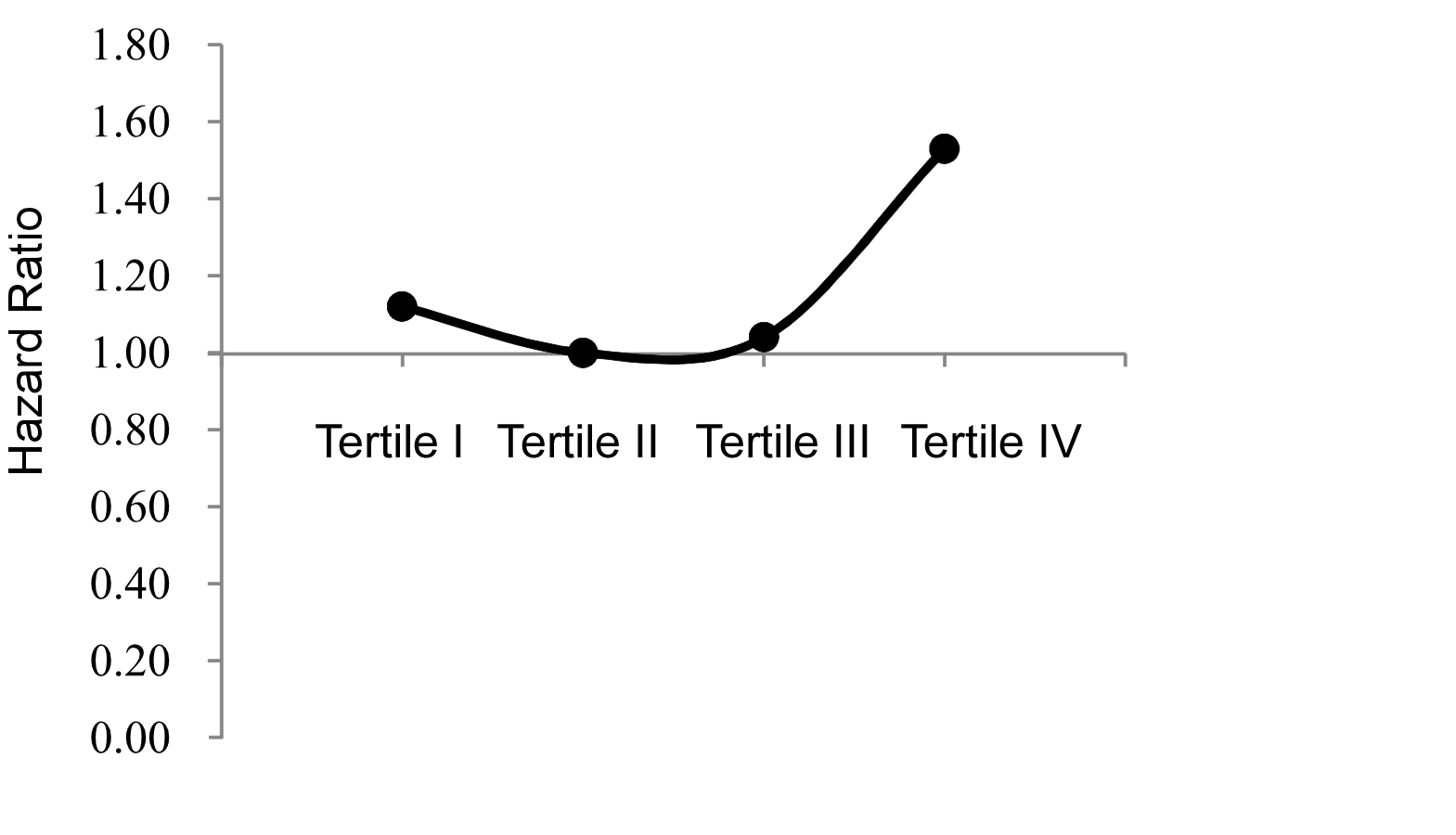

Supplement: S1 Fig — Compared with the Tertile II (≥1.09-≤1.48 mmol/L) as a reference, hazard ratios of stroke were 1.12 (95% confidence interval [CI], 0.88–1.41) for Tertile I (<1.09 mmol/L), 1.04 (95% CI, 0.90–1.28) for Tertile III (>1.48-≤1.90 mmol/L), and 1.53 (95% CI, 1.15–2.18) for Tertile IV (>1.90 mmol/L). Adjusted for age, sex, BMI, comorbidities, hemoglobin, serum albumin, albumin-corrected calcium, total cholesterol, hs-CRP, 24-h urine output, residual kidney function. (TIF) [file pone.0140887.s001.tif]
