# Supplementary material for: Prevalence and Impact on Stroke in Patients Receiving Maintenance Hemodialysis versus Peritoneal Dialysis: A Prospective Observational Study
Source: PLoS One. 2015 Oct 20;10(10):e0140887. doi: 10.1371/journal.pone.0140887 (PMC4617449; doi:10.1371/journal.pone.0140887)
Supplement: S1 Table — (DOCX) [file pone.0140887.s002.docx]

**S1 Table.** The Cox regression analysis of triglycerides for stroke in all dialysis patients

| Total triglycerides (mmol/L) | Crude HR (95% CI) |  | Adjusted HR (95% CI) ^a^ |
| --- | --- | --- | --- |
| < 1.09 | 1.10 (0.93-1.32) |  | 1.12 (0.88-1.41) |
| ≥ 1.09 - ≤1.48 | 1.00 |  | 1.00 |
| > 1.48- ≤ 1.90 | 1.05 (0.88-1.22) |  | 1.04 (0.90-1.28) |
| > 1.90 | 1.48 (1.07-1.96) |  | 1.53 (1.15-2.18) |

^a^ Adjusted for demographics (age, sex, BMI), comorbidities (diabetes, hypertension, cardiovascular disease), laboratory variables (hemoglobin, serum albumin, albumin-corrected calcium, total cholesterol, hs-CRP, 24-h urine output, residual kidney function).
